# Studies on the Regulation and Molecular Mechanism of Panax Ginseng Saponins on Senescence and Related Behaviors of *Drosophila melanogaster*

**DOI:** 10.3389/fnagi.2022.870326

**Published:** 2022-06-17

**Authors:** Qiushi Zhao, Ying Liu, Siyu Zhang, Yuchu Zhao, Chenxi Wang, Keqiang Li, Zecheng Jin, Juhui Qiao, Meichen Liu

**Affiliations:** ^1^School of Life Sciences, Jilin University, Changchun, China; ^2^Jilin Ginseng Academy, Changchun University of Chinese Medicine, Changchun, China; ^3^School of Pharmaceutical Sciences, Changchun University of Chinese Medicine, Changchun, China

**Keywords:** total ginsenosides, aging, healthy lifespan, transcriptome analysis, mechanism

## Abstract

In an increasingly aged global population, achieving healthy life expectancy through natural and safe drug interventions is highly desirable. Here we show that total ginsenosides (TGGR), the main active components in the traditional Chinese medicine, ginseng, promote longevity across species. In *Drosophila*, an intriguing effect of TGGR on lifespan was the relatively narrow treatment window to elicit long-term benefits. TGGR administration during early adulthood, and especially during midlife, was sufficient to extend lifespan in both sexes. TGGR did not increase lifespan by reducing food intake or reproductive capacity; rather, TGGR increased the fertility of male *Drosophila*. TGGR augmented healthspan readouts associated with youth and with healthy aging, such as motility, intestinal barrier integrity, and biorhythm homeostasis. TGGR treatment also improved some types of stress resistance in both sexes, including increased tolerance to starvation and oxidation, and shifting “aged” gene expression patterns toward “healthy” patterns seen in the young. Gene expression, pharmacological and genetic epistatic analyses demonstrated that TGGR effects require normal expression of genes involved in insulin, TOR and MAPK signaling. The positive effects of TGGR on both healthspan and lifespan, coupled with its mechanism of action via evolutionarily conserved signaling pathways, demonstrate it to be a promising anti-aging drug.

## Introduction

The population of the world is becoming increasingly aged. It is predicted that the world’s older adult population will reach a peak of 2.1 billion in 2050, which will pose a threat to social and economic stability (Seals et al., 2016). Given this projection, it is of the upmost importance to understand the aging process and to be able to intervene in diseases for which aging is the main risk factor. Aging is a process in which the physiological functions of various systems and different structural levels of an organism decline with increasing age. There are many theories about the mechanisms that underlie aging, but few there are effective treatment options that affect aging. In recent years, plant compounds that have anti-aging effects have been intensively studied (Wu et al., 2022; Zhang et al., 2022). Such compounds are derived from the natural environment and have low toxicity and few side effects and, therefore, have great promise in the development of drugs to treat age-related diseases.

The anti-aging activities of *Panax ginseng* C. A. Meyer (ginseng) have been documented in the “Huang Di Nei Jing,” the earliest compilation of Chinese medicine written more than 2,000 years ago. Many studies have shown that ginsenosides, the main active components in ginseng, have a positive effect on delaying aging and can effectively improve age-related cardiovascular disease, neurodegenerative diseases such as Parkinson’s disease and Alzheimer’s disease, as well as tumors (Tangrodchanapong et al., 2020). These actions are closely related to their anti-inflammatory and anti-oxidant effects and effects on mitochondrial homeostasis (Hyun et al., 2022). Therefore, ginsenosides have the potential to be used as an alternative natural medicine for anti-aging and life-prolonging effects. A recent study reported that total ginsenoside extract can prolong the lifespan and healthy lifespan of *Caenorhabditis elegans* (*C. elegans*) by activating anti-oxidation signaling pathways (Wang et al., 2021). However, the use of ginsenoside extract for delaying human aging, its anti-aging activities, optimal dosage and means of administration in different species need further investigation, and its multi-target mechanism of action needs to be elucidated.

Here, we used different systems, including *Drosophila melanogaster* (*Drosophila*), *C. elegans*, and human diploid fibroblasts (MRC-5 cells) to systematically evaluate the anti-aging effect of total ginsenosides of ginseng root (TGGR). We established a comprehensive system to characterize *Drosophila* health status to evaluate the concentration and optimal intervention period and to understand the effects of TGGR on aging-related systemic changes and stress resistance. Transcriptome sequencing and bioinformatics analysis was used to predict the multi-action targets of TGGR and RNA interference was used to identify genes and processes involved in *Drosophila* lifespan extension by TGGR. These data indicate an evolutionarily conserved anti-aging effect of TGGR and provide a comprehensive dataset for investigating the underlying mechanisms of ginseng in modulating lifespan.

## Materials and Methods

### Cell Subculture of MRC-5

MRC-5 cells were obtained from the American Type Culture Collection (Rockville, United States) and cultured in MEM complete medium (supplemented with 10% fetal bovine serum and 1% penicillin-streptomycin) at 37°C in a 5% CO_2_ atmosphere. Cell density was 4.0 × 10^5^/mL when the cells were cultured, and each culture flask was inoculated with 1 mL. Drug intervention has been carried out since the PDL7 generation. If the cells cannot grow to the bottom of the bottle within 3 weeks, these cells are defined as the last generation of cells.

### Cell Viability by CCK-8 Assay of MRC-5

Take PDL7 MRC-5 cells (2.0 × 10^5^ cells/mL) for assay. TGGR stock solution (1 mg/mL in PBS), diluted with complete medium to a final concentration of 40, 80, 120, 160, 200, 240, 280 μg/mL. According to the CCK-8 kit (Boster Bioengineering Co., Ltd., Wuhan, China) operating instructions, add 20 μL of detection solution to each well, and place it in the dark at 37°C for a period of time, and then measure the absorbance value (450 nm) of each well.

### β-Galactosidase Staining Assay of MRC-5 Cells

Take MRC-5 cells of PDL28 (4.0 × 10^5^ cells/mL) for assay. Follow the SA-β-gal staining kit (Biyuntian Biotechnology Co., Ltd., Shanghai, China) for cell fixation and cell staining, observe and take pictures under a microscope, and count the proportion of positive cells (cells are stained blue).

### Lifespan Analysis of *Caenorhabditis elegans*

Use NGM solid medium to carry out the life test, and refer to for the specific method (Admasu et al., 2018). Use a sterile self-made insect picker to pick 120 L4 nematodes into NGM petri dishes (with or without drugs) and record the day as day 0. The nematodes were transferred to a new NGM petri dish every day afterward, and the nematodes deaths were counted every other day until the late reproductive period (usually the 4th day). The criteria for judging the death of nematodes are no response to mechanical stimulation and no swallowing.

### *Drosophila* Stocks, Husbandry, and Lifespan Analysis

The wild-type stock Canston-S was collected from Dr. Yufeng Yang (Institute of Life Sciences, Fuzhou University, Fuzhou, China). 4E-BP-RNAi (CG8846), and S6K-RNAi (CG10539) were obtained from the Bloomington *Drosophila* Stock Center. Akt-RNAi (CG4006) was obtained from the Vienna *Drosophila* RNAi Center. To eliminate genetic background, RNAi lines were backcrossed into Canton-S for six generations. The males of all RNAi flies were crossed with da-GS-GAL4 virgin females, F1 generation females were collected, transferred to food containing 20 μM RU486 for activation. RU486 (20 μM) was added into the food after cooling it to 50°C. TGGR (Total Ginsenoside Ginseng Root, Shanghai Yuanye Biotechnology Co., Ltd., Shanghai, China) in ddH_2_O were added to sugar-yeast-agar (SYA) medium to a final concentration of 0.5, 2.5, and 5 mg/mL. Equivalent volumes and concentrations of vehicle were added to SYA medium for control treatments. Female and male flies were sorted onto SYA medium or contains drugs medium that was replaced every 1–2 days throughout life. Flies were cultured in a humidified (60% relative humidity), temperature (26–27°C) controlled incubator with a 12 h on/off light cycle.

### *Drosophila* Food Intake, Fecundity, and Triglyceride Measurements

Food intake were measured as previously described (Staats et al., 2019). Fecundity was quantified as number of eggs laid within 24 h, and triglyceride measurements were performed as previously described (Niraula et al., 2018).

### *Drosophila* Smurf Assay

In order to investigate TGGR effects on such a phenomenon, flies were aged on SYA medium supplemented or not with TGGR until the day of the “Smurf” assay, in which dyed medium was prepared using standard medium with blue dye #1 (Soleibao Technology Co., Ltd., Beijing, China) added at a concentration of 2.5% (w/v). Flies were kept on dyed medium overnight. A fly was counted as a Smurf when dye coloration could be observed outside of the digestive tract.

### *Drosophila* Assays of Locomotor Activity

On the day of assay, flies were transferred to new fly glass pipette (5 × 65 mm) that contain fresh diets. Then, the pipette was placed into the DAM2 *Drosophila* Locomotor Activity Monitor (Trikinetics, United States) and data were acquired and processed with DAM System 310 software (Trikinetics, United States). The recording signal interval to record once every 5 min, and the light cycle is 12 h on/off for 24 h. The circadian activity rhythm behavior curve is drawn with Excel.

### *Drosophila* Negative Geotaxis (Climbing) Assay

On the day of assay, flies were transferred with CO_2_ anesthesia into glass pipette (height 22 cm, diameter 1.0 cm, capacity 25 mL) capped with cotton plug, and after 15 min of awakening, the glass pipette is placed vertically for measurement. In the experiment, all flies were shaken down to the bottom of the tube, and the data was recorded by taking pictures at 5 s. Climbing index evaluation method: reserve 18 cm from the bottom of the test tube to the lower end of the tampon as the flies movement area, which was divided into 1, 2, 3, 4, 5, 6, 7, 8, 9 from the bottom to the top (the corresponding score for the area was 1, 2, 3, 4, 5, 6, 7, 8, 9 points) There were 9 areas in total, and the climbing index is the sum of the points of each area.

### *Drosophila* Starvation, Desiccation and Oxidative Stress Assays

Sorted flies were fed on SYA or SYA supplemented with TGGR until the day of assay, files were transferred to medium made of 1% agar (starvation assay) or to empty vials (desiccation assay) or to supplemented with SYA diets contain 5% (w/v) H_2_O_2_ (H_2_O_2_ stress assay) or to supplemented with SYA diets contain 6 mg/mL SDS (SDS stress assay). Construct survival curves after recording dead flies every 12 h (starvation and SDS assays) or every 2 h (H_2_O_2_ stress assay) or every 1 h (desiccation assay). Replace flies daily with fresh starvation or SYA/SDS supplemented medium and eliminate dead flies.

### *Drosophila* Weight Assay

Keep the flies in a pre-weighed centrifuge tubes and the total weight was measured at the day of weight assay. Final weight of flies was calculated by subtracting pre-measured weight of centrifuge tubes.

### *Drosophila* Antioxidation Assay

Frozen *Drosophila* samples (pre-weighed) were homogenized in physiological saline and centrifuged at 3,000 rpm for 10 min at 4°C. Superoxide dismutase (SOD) and catalase (CAT) were detected according to the instructions of the assay kit (Nanjing Jiancheng Bioengineering Institute, Nanjing, China).

### High Performance Liquid Chromatography Analysis of Total Ginsenosides of Ginseng Root

Reference substance: Ginsenoside Rg1, Re, Rf, Rg2, Rb1, Ro, Rc, Rb2, Rb3, Rd, purity: 98.10, 98.40, 98.00, 97.80, 98.81, 97.90, 98.00, 97.80, 97.60, and 92.10%, the batch numbers were 22427-39-0, 52286-59-6, 52286-58-5, 52286-74-5, 41753-43-9, 34367-04-9, 11021-14-0, 11021-13-9, 68406-26-8, 52705-93-8, all purchased from Le Mei Tian Pharmaceutical Co., Ltd.

Use HLPC to analyze the content of monomeric saponins contained in TGGR. The analysis method refers to the literature (Yao et al., 2016). The specific operation is as follows:

Chromatographic conditions: Agilent EC-C18 chromatographic column (4.6 mm × 150 mm, 2.7 μm); mobile phase acetonitrile (A) −0.1% phosphoric acid aqueous solution (B); column temperature: 40°C; flow rate 1 mL/min; injection volume: 10 μL; detection wavelength: 203 nm; gradient elution: (0–23 min, 18–21%A; 23–35 min, 21–28%A; 35–80 min, 28–32%A).

Preparation of mixed reference solution: precision weighing reference, add methanol to make ginsenoside, Rg1, Re, Rf, Rg2, Rb1, Ro, Rc, Rb2, Rb3, Rd, the mass concentrations were 1.38, 1.46, 1.50, 0.81, 0.69, 1.33, 1.38, 1.36, 1.60, 1.54 mg/mL mixed reference substance stock solution.

Preparation of test solution: precisely weigh the total ginsenoside sample and add 70% methanol, the concentration was 2.95 mg/mL, ultrasonic extraction (500 Hz) 30 min. Make up the weight after letting it cool, and pass through a 0.22 μm filter membrane.

### Transcriptome Analysis

Transcriptome analysis was performed using six samples of both sexes from young control flies (7 days), aged model (40 days), and aged TGGR-treated flies (40 days).

To extract the genes whose expression was reduced with aging and recovered by TGGR administration, we selected the genes for which the expression in the aged model flies less than that in the young control flies, and those for which the expression in the aged TGGR-treated flies was greater than that in the aged models. In addition, to extract the genes whose expression increased with aging and was reduced by TGGR administration, we selected the genes for which the expression in the aged models was greater than that in the young control flies, and those for which the expression in the aged TGGR-treated flies was less than that in the aged models. All the differential genes of the fold changes were not less than 2.0 [| log_2_(Fold Change)| > 1] and with an adjusted *Q*-value less than 0.001. The princomp function in R software was used for PCA analysis. KEGG pathway enrichment analysis of differential genes was used to calculate biologically significant pathways using phyper function in R software,^1^ and the results of KEGG enrichment analysis were displayed in scatter plots drawn by Origin software (Version No. 9.1, OriginLab corporation, Massachusetts, United States). KEGG enrichment was measured by Rich Factor, *Q*-value and the number of genes enriched in this pathway. All sequence data were submitted to the Sequence Read Archive (accession number: PRJNA806668) and are freely available at the NCBI.^2^

### *Drosophila* Quantitative RT-PCR Analysis

Flies were starved for 2 h and stored at −80°C. Total RNA was extracted using the TRIzol reagent (Invitrogen, Carlsbad, CA, United States) and then synthesized into cDNA. Rp49 expression was used as the internal control. Gene expression was calculated using the 2^–ΔΔCT^ method (Dong et al., 2019). The average of at least three repeats was used for each biological data point, and the sequences of all the primers are listed in Supplementary Table 1.

### Western Blot Analysis

*Drosophila* were harvested and snap-frozen after aging and drug treatment, and tissue was homogenized and normalized for protein content using lysate (Ratliff et al., 2016). Antibodies used were as follows: Anti-phospho-Thr398-S6K (Cell Signaling #9209, United States); anti-S6K (Cell Signaling #9202, United States); anti-phospho-Thr308-Akt (Thermo Fisher Scientific 44-602G, United States); anti-Akt (Cell Signaling #9272, United States); anti-phospho-Thr37/46-4E-BP1 (Cell Signaling #2855, United States);anti-4E-BP1 (Cell Signaling #9452, United States); anti-ERK1/2 (Cell Signaling #4695, United States); anti-phospho-Thr202/Tyr204-ERK1/2 (Cell Signaling #9101, United States); anti-beta Tublin (abcam ab179513, Britain); horseradish peroxidase conjugated anti-mouse IgG (Cell Signaling #7076, United States) or horseradish peroxidase-conjugated anti-rabbit IgG (ProteinTech SA00001-2, United States). Nitrocellulose filter membranes with attached protein bands were imaged using the iBright FL1000 Imaging System (Invitrogen; Thermo Fisher Scientific, Inc., United States). Image J software 1.53a (National Institutes of Health, United States) was used to quantify the gray value of western blot bands and protein expression level was normalized as the gray value of the target protein/the loading control protein.

### Statistical Analysis

Data were expressed as the means ± standard error of mean (SEM). Statistical analysis was performed with GraphPad Prism (Version No. 6, GraphPad Software, La Jolla, CA, United States). Statistical significance was established using one-way ANOVA followed by Dunnett’s *t*-test except survival. Survival among groups were compared and tested for significance with a log-rank test. *p* ≤ 0.05 was considered to indicate a statistically significant difference.

## Results

### Total Ginsenosides of Ginseng Root Promotes Longevity Across Species

**FIGURE 1:**
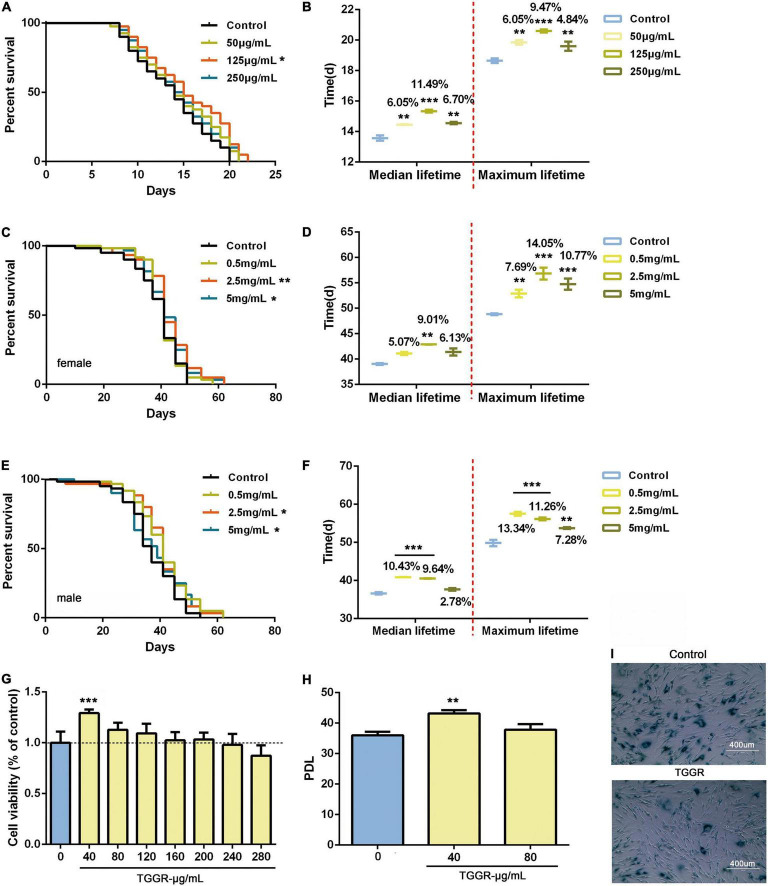
TGGR promotes longevity in *C. elegans, Drosophila* and human cells. Survival of nematodes **(A,B)**, flies **(C–F)**, and MRC-5 cells **(G–I)** during aging with supplementation of medium with TGGR. Representative survival curve **(A)** and median and maximal lifespan **(B)** in nematodes (*N* = 80 nematodes, one-way ANOVA). Survival curve and associated pairwise log-rank tests in female **(C)** and male flies **(E)**. The median and maximal lifespan in female **(D)** and male flies **(F)** (*N* = 120 flies, two-tailed unpaired *t*-test). **(G)** CCK-8 assays to detect the effect of TGGR on the viability of MRC-5 cells (*N* = 6, one-way ANOVA). **(H)** Effect of TGGR on the population doubling level (PDL) of MRC-5 cells [PDL = 3.32 (logN2-logN1) + X, where “N2” is the number of harvested cells of this generation, “N1” is the number of inoculated cells of the previous generation, and “X” is the PDL of the previous generation of cells] (*N* = 3, one-way ANOVA). **(I)** Aging-related galactosidase staining (SA-β-gal). Senescent cells are stained dark blue-green, and have increased cell volume and irregular shapes. *p < 0.05, **p < 0.01, ***p < 0.001 vs. the control.

We first examined whether TGGR can act as an anti-aging compound in two multicellular organisms, namely, *C. elegans* and *Drosophila*. TGGR treatment (125 μg/mL for nematodes and 2.5 mg/mL for flies) prolonged the median lifespan of both model organisms. In nematodes, chronic TGGR treatment extended median lifespan by 11.49% and maximum lifespan by 9.47% (Figures 1A,B). In female flies chronic TGGR treatment extended median lifespan by 9.01% and maximum lifespan by 14.05% (Figures 1C,D), while in male flies median lifespan was extended by 10.43% and maximum lifespan by 13.34% (Figures 1E,F). We also showed that TGGR extended the lifespan of flies to a greater degree compared with ginseng polysaccharide and ginseng total protein (Supplementary Figure 1).

To confirm and extend our results, we tested the effects of TGGR on human MRC-5 cells at population doubling level (PDL) 7. TGGR (40 μg/mL) had a significant effect on cell proliferation without toxicity in the range 0–240 μg/mL (Figure 1G). TGGR significantly increased the number of passages of MRC-5 cells (Figure 1H). Old cells were defined as PDL 28 (Gao et al., 2019), and aging-related galactosidase staining showed that the proportion of stained cells decreased after TGGR intervention (Figure 1I). These data indicate that TGGR augments the lifespans of evolutionarily diverse species from different phyla.

### Effects of Total Ginsenosides of Ginseng Root Are Duration and Age Specific

**FIGURE 2:**
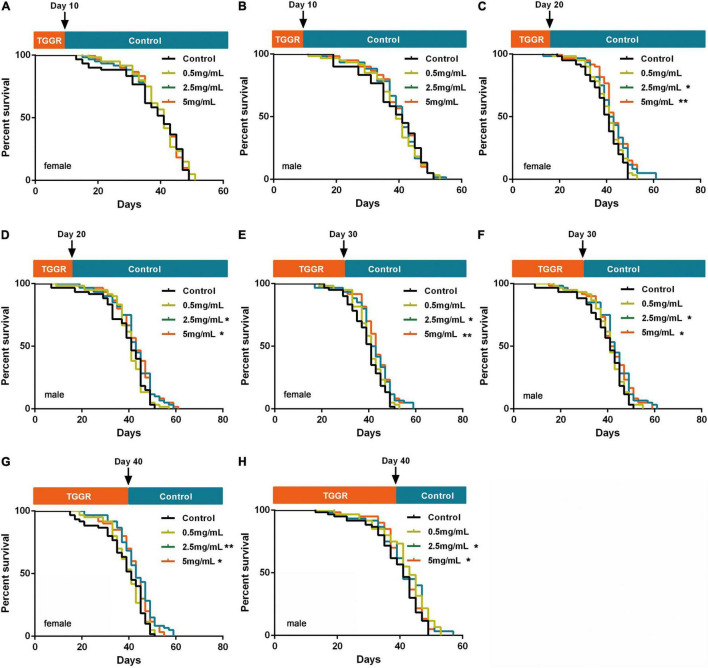
Screening of TGGR treatment duration. Short-term treatment with TGGR for 10 days early in adulthood of female **(A)** and male **(B)** flies. Medium-term treatment with TGGR for 20 days in female **(C)** and male **(D)** flies. Treatment with TGGR for 30 days in female **(E)** and male **(F)** flies. Long-term treatment with TGGR for 40 days in female **(G)** and male **(H)** flies. *N* = 120 flies per condition, Log-rank (Mantel-Cox) test. *p < 0.05, **p < 0.01 vs. the control.

A lifetime of continuous drug use is not realistic; therefore, we investigated the optimum duration and age for TGGR administration for extension of lifespan in flies. Ten days of TGGR treatment did not extended lifespan (Figures 2A,B), whereas 20 days (Figures 2C,D), 30 days (Figures 2E,F), and 40 days (Figures 2G,H) of TGGR treatment did. Lifespan extension was slightly reduced by TGGR treatment for 40 days compared with that for 20 and 30 days. Therefore, 20 days of continuous TGGR intervention was chosen as the optimum duration.

**FIGURE 3:**
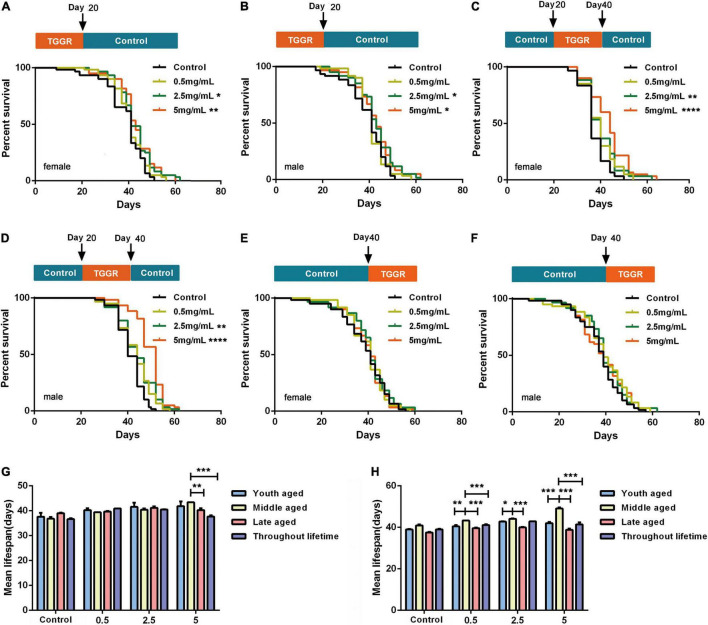
TGGR effect on *Drosophila* longevity. Brief treatment with TGGR for 20 days early in adulthood extended the lifespan of female **(A)** and male **(B)** flies; TGGR extended the lifespan of 20-day-old female **(C)** and male **(D)** flies at concentrations from 0.5 to 5 mg/mL (at 40 days, medium was changed to sugar-yeast-agar); Treatment with TGGR for 20 days in late-aged flies (TGGR medium at 40–60 days) did not significantly extend the lifespan of female **(E)** or male **(F)** flies [*N* = 120 flies per condition, Log-rank (Mantel-Cox) test]. Comparative analysis of the median lifespan of the four methods of administration in female **(G)** and male flies **(H)**; between 0 and 20-day-old (young-aged) flies, between 20 and 40-day-old (middle-aged) flies, and between 40 to 60 days (late-aged) flies (*N* = 120 flies per condition, two-way ANOVA). *p < 0.05, **p < 0.01, ***p < 0.001, ****p < 0.0001 vs. the control.

We next continuously exposed flies to TGGR for 20 days at different age stages: early (0–20 days), middle (20–40 days), and late (40–60 days) adulthood. Treatment of early and middle-aged flies with 5 mg/mL TGGR had a significant life-prolonging effect, but low-dose (0.5 mg/mL) TGGR did not. These two administration phases showed similar dose-dependent effects. Early TGGR treatment (5 mg/mL) extended median lifespan by 10.14% in female flies and by 7.64% in male flies (Figures 3A,B). Middle TGGR treatment (5 mg/mL) extended median lifespan by 15.24% in female flies and by 16.59% in male flies (Figures 3C,D). Late TGGR treatment did not extend lifespan (Figures 3E,F) and middle TGGR treatment extended lifespan the most (Figures 3G,H). Based on these results, there appears to be a duration and age specific window in which TGGR can exert its beneficial effects.

### Total Ginsenosides of Ginseng Root Extends Healthy Lifespan in *Drosophila*

**FIGURE 4:**
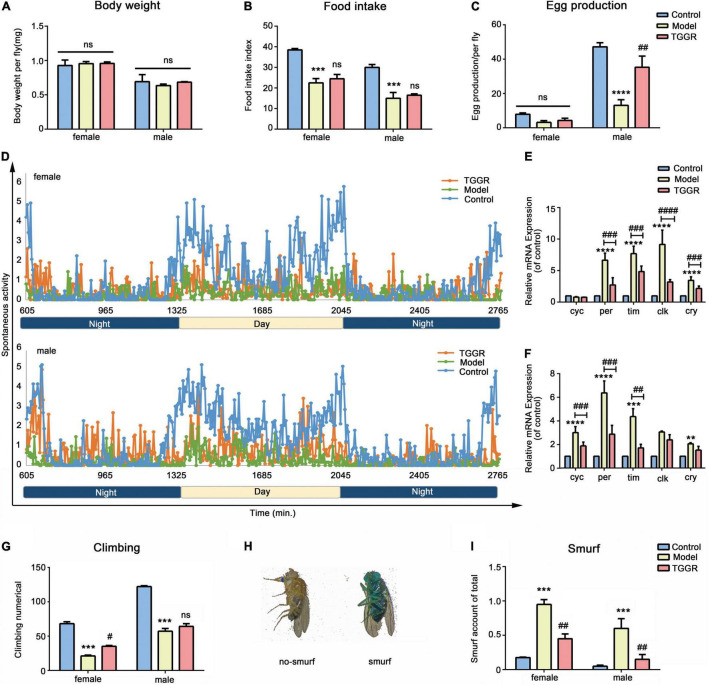
TGGR treatment prolongs healthspan. Body weight (**A**, *N* = 90 flies), feeding assay (**B**, *N* = 60 flies), fecundity (**C**, *N* = 60 flies), climbing numbers (**G**, *N* = 90 flies), intestinal integrity (**I**, *N* = 90 flies) with or without TGGR treatment. **(D)** Circadian rhythm of 40-day-old flies with or without TGGR treatment (*N* = 16 vials of one fly per condition). mRNA levels of biorhythm genes were analyzed in flies with or without TGGR treatment for 40 days (**E:** female; **F:** male). (*N* = 90 flies per condition, two-way ANOVA). **(H)** Representative images of ‘Smurf’ and ‘no Smurf’ in *Drosophila* “Control” was 7-day-old flies, “Model” was 40-day-old flies, and “TGGR” was 40-day-old flies with TGGR (5 mg/mL) treatment. ***p < 0.001, ****p < 0.0001 vs. the control; ^#^*p* < 0.05, ^##^*p* < 0.01, ^###^*p* < 0.001, ^####^*p* < 0.0001 vs. the model; ns means *p* > 0.05.

To investigate whether TGGR-mediated lifespan extension was related to enhanced healthspan, we examined various healthspan parameters in aged flies (40 day-old) following 20 days of TGGR treatment (continuously exposed from day 20 to 40). TGGR had no effect on body weight (Figure 4A). Reduced food intake can prolong longevity (Song et al., 2020); therefore, we tested whether TGGR treatment affects feeding behavior. We observed no significant change in this behavior with TGGR treatment (Figure 4B). Several interventions that promote longevity are frequently related to reproductive tradeoffs (He et al., 2021). Nevertheless, TGGR increased fecundity in aged male flies (Figure 4C). TGGR treatment produced a significant improvement in circadian rhythm (Figure 4D). Importantly, increased physical activity in the day-time eliminated restlessness at night time. Monitoring of circadian rhythm behavior found that TGGR regulated the biological rhythm of flies. The expression of *cyc* in female flies did not change, but *per*, *tim*, *clk*, and *cry* all showed an upward trend in expression in aging flies. TGGR ameliorated disrupted biological rhythm caused by aging to varying degrees (Figures 4E,F). Moreover, it delayed climbing decline at two concentrations that extend lifespan (Figure 4G).

Organ dysfunction is driven by aging, for example, intestinal barrier function, an evolutionary conserved biomarker (Rodriguez-Fernandez et al., 2019). To determine whether TGGR contributes to the restoration of gut function, we considered the presence of unabsorbed dye outside the *Drosophila* digestive tract as an indicator of loss of gut barrier function. There was significantly fewer “Smurf” (whole body dyed blue) in TGGR-treated (5 mg/mL) flies than in untreated flies, confirming enhanced barrier integrity in TGGR-treated flies (Figures 4H,I). In addition, TGGR can prolong the life span of flies that were acutely injured by sodium dodecyl sulfate (SDS) (Supplementary Figure 2). Collectively, these data demonstrate that TGGR treatment improves healthspan and modulates tissue homeostasis associated with aging.

### Total Ginsenosides of Ginseng Root Increased Resistance of *Drosophila* to Stress Conditions

The ability to withstand extrinsic stress is a marker of organismal health that declines with age. The effect of TGGR on various stresses, including, starvation, oxidative and heat shock stress, was analyzed in flies.

**FIGURE 5:**
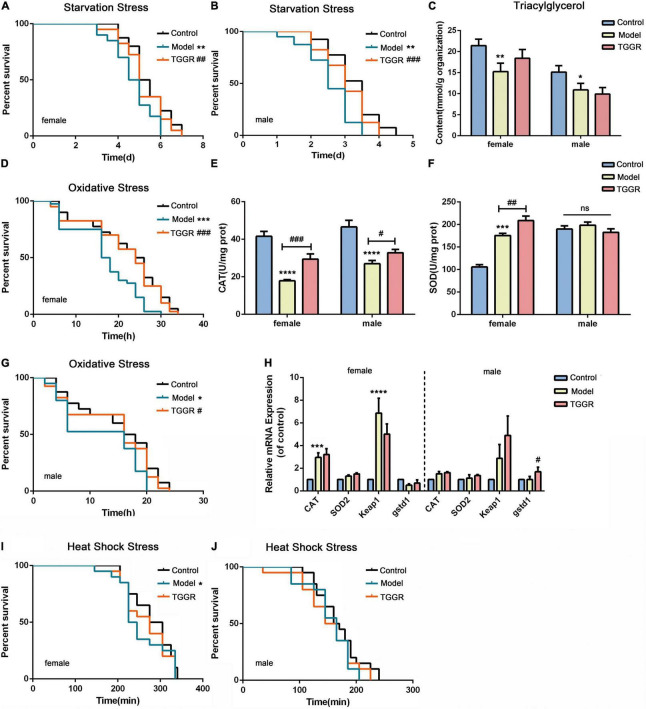
Effect of TGGR on stress resistance in *Drosophila*. Survival of flies under starvation stress (**A**, female; **B**, male), oxidative stress (**D**, female; **G**, male), and heat-shock stress (**I**, female; **J**, male) with or without TGGR treatment (*N* = 30 flies in per condition, two-tailed unpaired *t*-test). TAG levels in flies with or without TGGR treatment **(C)**; Enzyme activity of Cat **(E)** or SOD **(F)** with or without TGGR treatment; Analysis of anti-oxidant gene expression in flies by qRT-PCR with or without TGGR treatment (**H:** the left side of the dotted line indicates females and the right side, males) (*N* = 60 flies, two-tailed unpaired *t*-test). “Control” was 7-day-old flies, “Model” was 40-day-old flies, and “TGGR” was 40-day-old flies with TGGR (5 mg/mL) treatment. *p < 0.05, **p < 0.01, ***p < 0.001, ****p < 0.0001 vs. the control; ^#^*p* < 0.05, ^##^*p* < 0.01, ^###^*p* < 0.001 vs. the model.

Resistance to starvation stress was significantly increased by TGGR (Figures 5A,B, female, 10.84% increase; male, 15.60% increase). The storage of fat correlates with survival under starvation conditions; therefore, we examined the quantity of the lipid-storage molecule, triglyceride (TAG), in *Drosophila*. Aging reduced TAG levels compared with young flies (Figure 5C, female, 28.86% decrease; male, 27.90% decrease); however, no decrease in TAG levels was found with TGGR treatment in females or males. Under H_2_O_2_ oxidative stress conditions, TGGR enhanced survival in both sexes (Figure 5D, female, 21.94% increase; Figure 5G, male, 18.57% increase). Next, we examined anti-oxidant enzyme activities and related gene expression levels to confirm the anti-oxidant benefits of TGGR. The enzyme activities of catalase (Cat) (Figure 5E) and superoxide dismutase (SOD) (Figure 5F) were elevated after TGGR treatment (except for SOD activity in male flies). Likewise, the mRNA levels of *Cat*, Mn-SOD (*Sod2*), kelch-like ECH-associated protein 1 (*keap1*) and *gstd1* were not significantly increased by TGGR treatment (Figure 5H, except for *gstd1* in male flies). No effect of TGGR on heat stress resistance (heat-shock) in females or males was observed (Figures 5I,J).

### Total Ginsenosides of Ginseng Root Leads to Pro-longevity Transcriptional Signatures in *Drosophila*

To determine how TGGR extends lifespan and healthspan, we used RNA sequencing (RNA-Seq) to compare transcription in whole flies at 7 days old (control), 40 days old (model), and 40 days old with TGGR treatment for 20 days (TGGR). Principal component analysis (PCA) demonstrated close agreement between replicate samples as well as the expression differences that could largely be attributed to aging or TGGR treatment (*X*-axis, 51.1%) (Supplementary Figure 3A). Interestingly, compared with the untreated flies, the transcriptome in the TGGR-treated flies showed a significant trend toward a younger pattern. The average reads per kilobase of transcript per million reads mapped (RPKM) values of expressed transcripts of each treatment group were checked by hierarchical cluster analysis, which further emphasized the similarity between duplicate samples and the influence of aging and TGGR treatment on global transcriptome trends (Supplementary Figure 3B).

**FIGURE 6:**
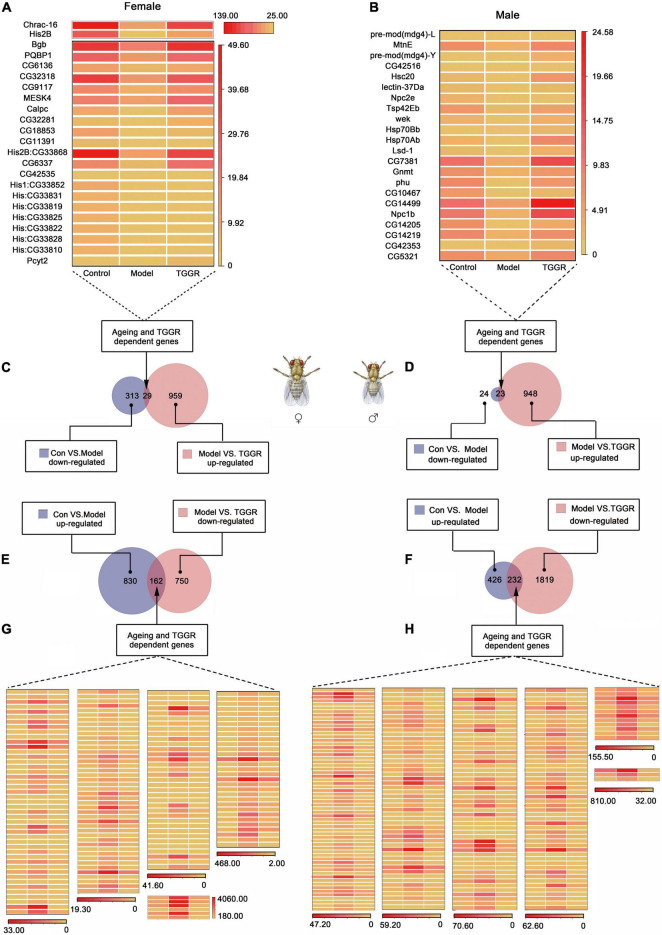
Analysis of differential gene expression. **(A,B)** and **(G,H)** Genes with aging- or TGGR-induced fold changes in expression are represented by heatmaps. **(C–F)** Venn diagrams of aging and TGGR regulation of differentially expressed genes (female, **A,C,E,G**; male, **B,D,F,H**). “Control” was 7-day-old flies, “Model” was 40-day-old flies, and “TGGR” was 40-day-old flies with TGGR (5 mg/mL) treatment.

Genes with a more than a 2.0-fold change in expression (with statistical significance) in response to aging or TGGR treatment are presented in Venn diagrams (Figures 6C–F) and shown in a table (Supplementary Figure 3C). In total, for aging, 705 genes (658 induced; 47 repressed) were altered in males and 1,334 genes (992 induced; 342 repressed) were altered in females. Following TGGR exposure, 29 repressed genes in females and 23 in males were up-regulated, while 162 induced genes in females and 232 in males were down-regulated. The differential expression of these genes is represented in a heatmap (Figures 6A,B,G,H). Genes expressed differentially in response to TGGR also showed a significant shift toward more youthful expression patterns.

### Total Ginsenosides of Ginseng Root Induced Changes in Gene Co-expression Networks

**FIGURE 7:**
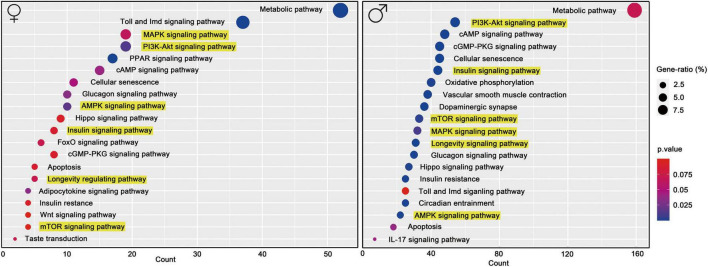
KEGG functional pathway analysis. KEGG pathway enrichment with aging and TGGR treatment.

Functional characteristics of differentially expressed genes were discovered using KEGG pathway enrichment analyses. Among the molecular pathways stimulated by TGGR, many are associated with longevity, including those of AKT, MAPK, TOR, FOXO, and insulin signaling, cellular senescence, apoptosis, oxidative phosphorylation, regulation of circadian rhythms, synaptic plasticity, and circadian entrainment (Figure 7).

To validate the results of the RNA-Seq analysis, we used qRT-PCR to analyze the expression of the most representative genes, including protein kinase B (*Akt*), phosphatidylinositol 3 kinase (*PI3K*), target of complex (*TORC*), 4E binding protein (*4E-BP*), and protein s6 kinase (*S6K*) (Supplementary Figure 4). Changes in these genes were similar to those seen by RNA-Seq analysis.

### Longevity-Regulating Signaling Pathways Are Required for Total Ginsenosides of Ginseng Root to Modify Lifespan

**FIGURE 8:**
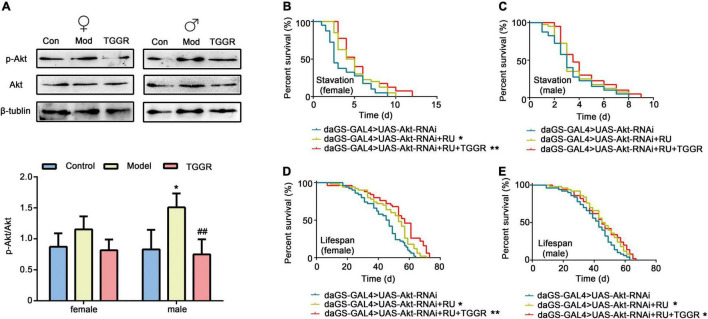
TGGR-mediated longevity is influenced by Akt, a component of the insulin pathway. **(A)** TGGR decreased the level of p-Akt (*N* = 50 flies per condition, two-tailed unpaired *t*-test). **(B,C)** Effects of Akt knockdown on starvation resistance effects in flies; **(D,E)** changes in lifespan of Akt knockdown flies [*N* = 60 flies per condition, Log-rank (Mantel-Cox) test]. “Control” was 7-day-old flies, “Model” was 40-day-old flies, and “TGGR” was 40-day-old flies with TGGR (5 mg/mL) treatment. *p < 0.05, **p < 0.01 vs. the control; ^##^*p* < 0.01 vs. the model.

The KEGG pathway enrichment analyses prompted us to examine the relationship between TGGR and longevity-regulating signaling pathways, especially insulin, AKT, MAPK, and TOR signaling pathways. To assess the link between the effect of TGGR on longevity and insulin, phosphorylation of the signaling intermediate, Akt, was first examined. Compared with the aging group, TGGR treatment decreased levels of p-Akt to those of the control (Figure 8A). We next tested whether TGGR-mediated lifespan extension was affected by this reduction in insulin and Akt knockdown flies. We found a marked increase in starvation resistance (Figures 8B,C; except in male starvation-resistant flies) and lifespan (Figures 8D,E). TGGR treatment was ineffective in Akt knockdown flies.

**FIGURE 9:**
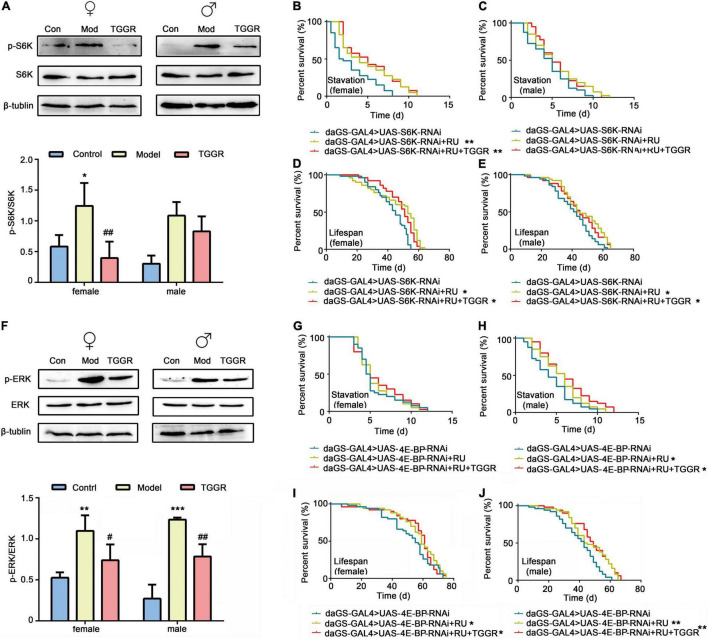
TGGR regulates TOR and MAPK pathways by affecting the major components, S6K, 4E-BP and ERK. **(A)** Phosphorylation levels of S6K were analyzed by western blotting (*N* = 50 flies per condition, two-tailed unpaired *t*-test). **(B,C)** Starvation resistance in S6K-knockdown flies. **(D,E)** Lifespan changes in S6K knockdown flies [*N* = 50 flies per condition, Log-rank (Mantel-Cox) test]. **(F)** Western blot analysis of ERK phosphorylation levels (*N* = 50 flies per condition, two-tailed unpaired *t*-test). **(G,H)** 4E-BP downregulates starvation resistance in flies. **(I,J)** Lifespan changes in 4E-BP knockdown flies [*N* = 50 flies per condition, Log-rank (Mantel-Cox) test]. “Control” was 7-day-old flies, “Model” was 40-day-old flies, and “TGGR” was 40-day-old flies with TGGR (5 mg/mL) treatment. *p < 0.05, **p < 0.01, ***p < 0.001 vs. the control; ^#^*p* < 0.05, ^##^*p* < 0.01 vs. the model.

Akt is a key effector of insulin and TOR signal transduction. Therefore, we next examined the effect of TGGR on the TOR pathway. Phosphorylation of ribosomal S6K, which lies downstream of TOR, was decreased in TGGR-treated flies (Figure 9A). To explore the target of TOR affected by TGGR treatment, we down-regulated S6K using RNAi, and starvation resistance of flies was not affected by TGGR treatment (Figures 9B,C). Otherwise, knockdown of S6K increased fly lifespan in non-starvation conditions and TGGR failed to extend it further (Figures 9D,E).

RNAi of 4E-BP in female flies had no significant effect on starvation resistance, but prevented starvation resistance exerted by TGGR treatment (Figures 9G,H). Similarly, TGGR did not prolong the lifespan of 4E-BP knockdown flies (Figures 9I,J). Taken together, normal output of the TOR pathway is required for TGGR to combat starvation tolerance and longevity.

ERK1/ERK2 is part of the MAPK pathway involved in the stress response and is known to regulate TOR signaling through phosphorylation in mammalian models. We therefore examined phosphorylation levels of ERK1/ERK2. TGGR did not significantly alter the total protein level of ERK but reduced the pERK/ERK ratio relative to controls (Figure 9F). These findings indicate that TGGR reduces MAPK signaling, which may in turn reduce TOR signaling.

### Analysis of Total Ginsenosides of Ginseng Root Components That Affect Longevity

**FIGURE 10:**
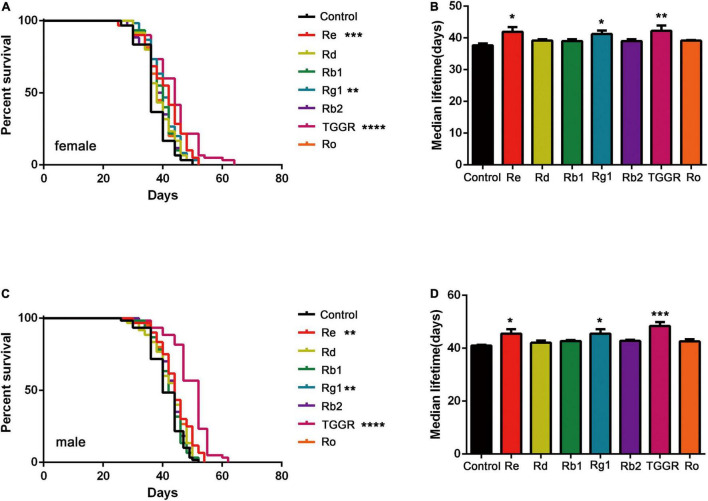
The effect of TGGR monomer components on longevity in *Drosophila.* Survival of female **(A)** or male **(C)** flies treated with TGGR or different ginsenosides [*N* = 60 flies per condition, Log-rank (Mantel-Cox) test]; The median lifespan of female **(B)** or male **(D)** flies treated with different ginsenosides (*N* = 60 flies per condition, One-way ANOVA). The flies were treated with the following drug concentrations: Re (300 μg/mL), Rd (500 μg/mL), Rb1 (300 μg/mL), Rg1 (500 μg/mL), Rb2 (300 μg/mL), TGGR (5 mg/mL), Ro (300 μg/mL). *p < 0.05, **p < 0.01, ***p < 0.001, ****p < 0.0001 vs. the control.

To identify the main ginsenosides in the TGGR used, high performance liquid chromatography (HPLC) was applied (Supplementary Figure 5). We quantified 10 ginsenosides (Rg1, Re, Rf, Rg2, Rb1, Ro, Rc, Rb2, Rb3, Rd), of which Rg1, Re, Rb1, Ro, Rb2, and Rd were the most abundant (Supplementary Table 2). The main components of TGGR that affect longevity in *Drosophila* were explored by assessing the effects of different ginsenoside monomers on lifespan. Re and Rg1 had significant longevity effects second only to TGGR during the optimal intervention window (female: Figures 10A,B; male: Figures 10C,D). We speculate that the main active ingredients in aging delay were Rg1 and Re.

## Discussion

Aging is the most important risk factor for all major chronic ailments, including cancer, cardiovascular and neurodegenerative diseases (Ma et al., 2022). However, behavioral and pharmacological interventions that promote health upon aging remain rare. Here we report anti-aging properties of natural TGGR from ginseng. TGGR administration extends the lifespan of *Drosophila* and *C. elegans*, and reduces the senescence of human cell cultures. The present data together with previous data from *C. elegans* shows that TGGR has the potential to promote longevity across species. Interestingly, TGGR did not improve lifespan by reducing reproductive capacity; in fact TGGR increased the fertility of male flies. TGGR augmented health span readouts associated with youth and healthy aging, such as motility, intestinal barrier integrity, and biorhythm homeostasis. TGGR treatment also improved resistance to certain kinds of stress in both sexes, such as tolerance to starvation and oxidation. Furthermore, TGGR shifted gene expression in aged flies toward “healthy” patterns seen in young flies. Importantly, in no assay did we find any detrimental side-effects. Thus, TGGR can increase both lifespan and healthspan in *Drosophila*.

A particularly interesting feature of the effect of TGGR on longevity is the relatively small therapeutic window for long-term benefits. TGGR administration during early adulthood, and especially during midlife, was sufficient to extend lifespan in both male and female flies, indicating a “memory” effect. If TGGR reduces the risk of acute and reversible mortality at a given age, early life treatment with TGGR is expected to have effects similar to dietary restriction (Kebbe et al., 2021). Conversely, early life TGGR treatment slowed the underlying aging process, and flies had progressively longer life with TGGR treatment. Importantly, this effect was maintained when treatment was initiated in midlife, indicating meaningful therapeutic properties, a phenomenon that should be subject to further investigation in mammals.

The increased resistance to stress is also potentially a direct consequence of TGGR administration. Intriguingly, male flies show the same resistance to starvation stress compared with females but have lower TAG levels, indicating a mechanism other than increased fat storage. In addition, female flies showed better resistance to acute oxidative stress than male flies after TGGR intervention. TGGR can significantly increase the activity of both CAT and SOD in female flies but only CAT in male flies. Also, TGGR only increased *Cat* mRNA expression in female flies. The Keap1-Nrf2 signal transduction pathway plays a highly conserved role in regulating oxidative stress (Yao et al., 2022). Under normal physiological conditions, Nrf2 binds to the cytoplasmic inhibitor, Keap1, and exists in the cytoplasm, maintaining low transcriptional activity (Luo et al., 2021). However, under oxidative stress, Nrf2 translocates to the nucleus, binds to Marf proteins to form a heterodimer, and then binds to anti-oxidant response elements (AREs) to activate the expression of anti-oxidation genes (Matsumaru and Motohashi, 2021). Oxidative stress increased the mRNA level of the oxidation-promoting factor, *keap1*, which was inhibited by TGGR intervention. This inhibition was also more pronounced in female flies. These results indicated that there may be gender differences in anti-oxidant capacity induced by TGGR. However, there was no significant difference in the effects of TGGR on the healthy lifespan of both sexes, indicating that there must be other regulatory pathways in addition to oxidative stress involved in the response.

Restoring disrupted gene expression and the normal activities of signaling pathways through drug intervention is the most promising way to improve healthy aging (Roichman et al., 2021). The transcription profile of TGGR-treated flies revealed that age- and TGGR-dependent expression in flies involved many processes already known to be associated with aging, mainly involving the insulin, TOR and MAPK pathways. In yeast, *C. elegans*, *Drosophila*, and mice, these three pathways regulate lifespan (Tang et al., 2019). In the present study, pharmacological and genetic epistatic analysis in *Drosophila* demonstrated that the effect of TGGR on aging required normal expression of genes involved in insulin, TOR and MAPK signaling. The phosphorylation of AKT in insulin-like signaling, S6 kinase (S6K) in TOR signaling, and ERK1/2 in MAP kinase (MAPK) signaling was reduced by TGGR treatment relative to controls. These findings were corroborated by knockdown of Akt and S6K, which increased fly lifespan in both starvation and non-starvation conditions; increases that TGGR was not able to extend. It should be mentioned that no significant changes were observed in the protein expression levels of Akt and S6K compared to the mRNA levels. It is speculated that the possible reasons are the difference between sensitivity of the two assays (qRT-PCR and WB) (Sroka et al., 2016), the decrease of mRNA stability due to aging and the possible indirect regulatory effect of TGGR at the transcriptional level. Knockdown of 4E-BP blocked TGGR treatment-induced starvation resistance and also did not alter the lifespan of 4E-BP knockdown flies. The positive effects of ginseng in coping with aging-related diseases have been reported to be mediated by insulin (Hahn et al., 2010). For example, total saponin from Korean red ginseng has life-extending effects, in addition to reducing thrombin-induced phosphorylation of PI3K and Akt (Hou et al., 2020). Finally, to elucidate the components of TGGR that might be responsible for the pro-longevity effects, we used HPLC to identify and quantify the ginsenoside compounds in TGGR. The ginsenosides Rb1, Rg1, Rd, Rb2, Ro, and Re were abundant in TGGR. Rg1 and Re exhibited similar anti-aging effects as TGGR in *Drosophila*. Consistent with other studies, Rg1 and Re are the major components of Panax ginseng, which prolongs the lifespan of *C. elegans* (Wang et al., 2021), and Rg1 prevents cognitive impairment and hippocampal senescence in a rat model of aging (Zhou et al., 2020). In future studies, specific anti-aging effects of single saponins should be clarified, and synergistic studies of each saponin based on clear molecular mechanisms should be examined.

## Data Availability Statement

The original contributions presented in this study are included in the article/Supplementary Material, further inquiries can be directed to the corresponding author/s.

## Author Contributions

QZ, JQ, and ML: conceptualization. KL and ZJ: methodology. YL and SZ: formal analysis. YZ and CW: data curation. QZ: writing– original draft preparation. JQ and ML: writing – review and editing. ML: project administration and funding acquisition. All authors have read and agreed to the published version of the manuscript.

## Conflict of Interest

The authors declare that the research was conducted in the absence of any commercial or financial relationships that could be construed as a potential conflict of interest.

## Publisher’s Note

All claims expressed in this article are solely those of the authors and do not necessarily represent those of their affiliated organizations, or those of the publisher, the editors and the reviewers. Any product that may be evaluated in this article, or claim that may be made by its manufacturer, is not guaranteed or endorsed by the publisher.

## Footnotes

1
https://en.wikipedia.org/wiki/Hypergeometric_distribution

2
https://www.ncbi.nlm.nih.gov/sra/PRJNA806668
