# Supplementary material for: Studies on the Regulation and Molecular Mechanism of Panax Ginseng Saponins on Senescence and Related Behaviors of Drosophila melanogaster
Source: Front Aging Neurosci. 2022 Jun 17;14:870326. doi: 10.3389/fnagi.2022.870326 (PMC9252430; doi:10.3389/fnagi.2022.870326)
Supplement: Supplementary file 1 [file Data_Sheet_1.doc]

Supplementary Material

# Supplementary Tables

**Table S1. Gene expression probes used for the quantitative real-time PCR analysis**

| **Gene** | **Sequence** |
| --- | --- |
| Rp49 | F: 5'- ATCGGTTACGGATCGAACAA-3'  R: 5'- GACAATCTCCTTGCGCTTCT-3' |
| PI3K | F: 5'-CCTAATCTGCCTGTTGCCCA-3'  R: 5'-ACTGAGTCGCTTCGTTTCGT-3' |
| AKt | F: 5'-GAGTCGTGTGCTCAAGTCCA-3'  R: 5'-TGCATCACAAAACACAGGCG-3' |
| TORC | F: 5'-GCGCCTCTACTTTGATAGGAC-3'  R: 5'-TATTGATGTCCAGCAGGCCG-3' |
| S6K | F: 5'-CATGCATTTGGAGCGTGAGG-3'  R: 5'-TCCTTGCACAGTCCGAAGTC-3' |
| 4E-BP | F: 5'-ACCCTCTACTCCACCACTCC-3'  R: 5'-GGAGTTTGGCTCAATGGGGA-3' |
| sod2 | F: 5'-TCTGAAGAAGGCCATCGAGT-3'  R: 5'-CAGTTTGCCCGACTTCTTGT-3' |
| cat | F: 5'-ATGCGGCTTCCAATCAGTTGAT-3'  R: 5'-CGAAGTGCGACATCTCATCCA-3' |
| keap1 | F: 5'-GCGCTCGTCAGCCCATTTT-3'  R: 5'-GGATGCGCATAATTCCTCTTCTT-3' |
| gstd1 | F: 5'-TGATCAATCAGCGCCTGTA-3'  R: 5'-GCAATGTCGGCTACGGTAAG-3' |
| per | F: 5'-GGGATCATATCGCACGTGGAC-3'  R: 5'-CTGCGGCCAATCAGGTCCTG-3' |
| tim | F: 5'-GCCTGGGCAATGAGCCATTC-3'  R: 5'-GAGGTGGAGGCTCTGACTGG-3' |
| cry | F: 5'-CCACCGCTGACCTACCAAA-3'  R: 5'-GGTGGAAGCCCAATAATTTGC-3' |
| clk | F: 5'-ATGATGACGCACGTCAGTTCGC-3'  R: 5'-TCGATGGTGTTCTCGGTGATGC-3' |
| cyc | F: 5'-AAGGAGCAGCTATCCTCACTGG-3'  R: 5'-GGTCTTAACGGGCAACATGGTC-3' |

**Table S2. Composition and content of ginsenosides**

| Composition | Percentage of content (%) | tR/min | Standard Purity |
| --- | --- | --- | --- |
| Rg1 | 5.20 | 23.65 | ≥98% |
| Re | 13.97 | 24.35 | ≥98% |
| Rf | 0.97 | 40.04 | ≥98% |
| Rg2 | 1.73 | 49.36 | ≥98% |
| Rb1 | 8.87 | 54.23 | ≥98% |
| Ro | 5.75 | 57.55 | ≥98% |
| Rc | 2.65 | 59.22 | ≥98% |
| Rb2 | 6.48 | 64.62 | ≥98% |
| Rb3 | 1.45 | 66.91 | ≥98% |
| Rd | 7.67 | 76.49 | ≥98% |

# Supplementary Figures


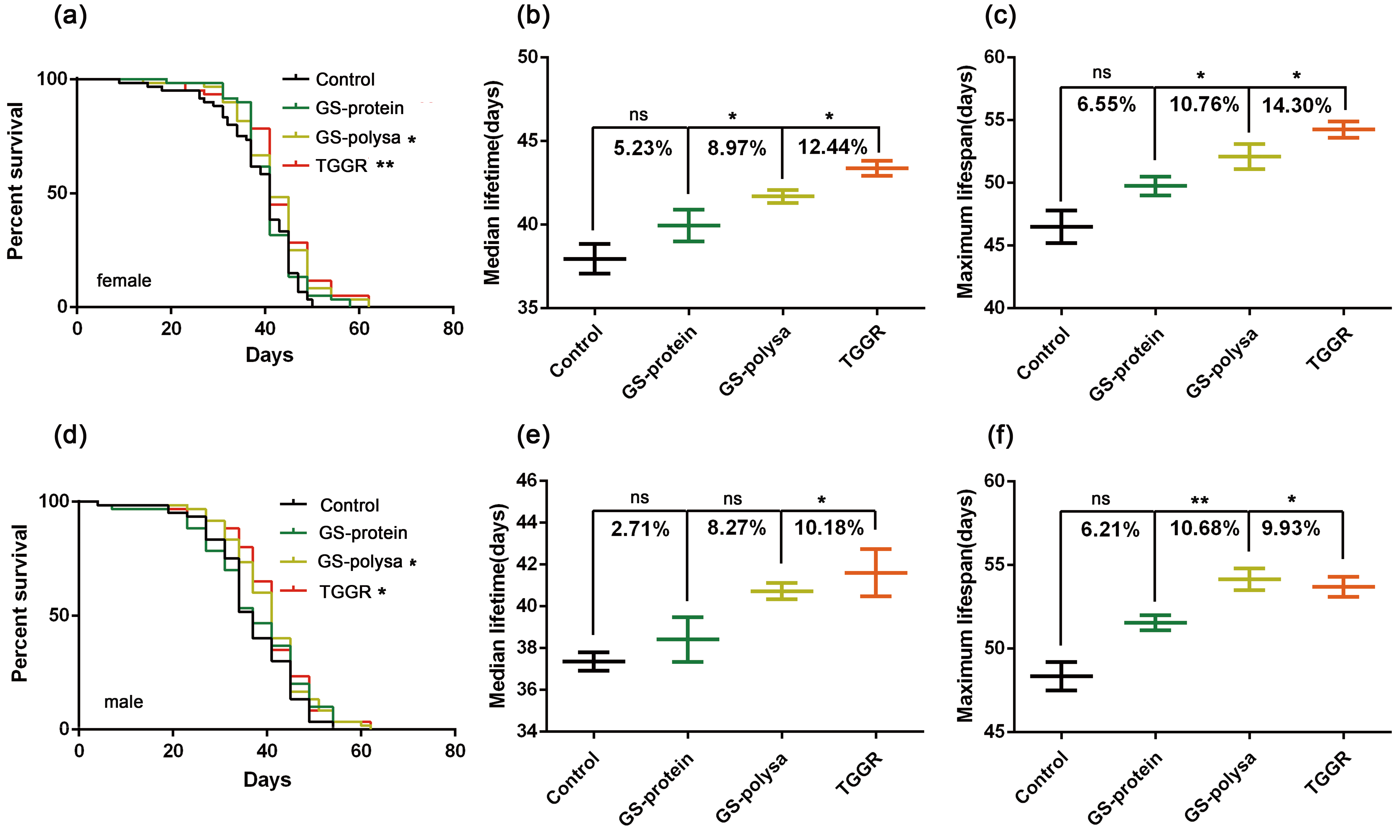


**Figure S1.** Main components of ginseng maximize longevity in *Drosophila*. Representative survival curve and associated pairwise log-rank tests in females (**a**) and males (**d**) (N = 120 flies per condition, Log-rank (Mantel-Cox) test). The midian (**b**) and maximal (**c**) lifespan in females. And the midian (**e**) and maximal (**f**) lifespan in males (N = 120 flies per condition, one-way ANOVA). **p* < 0.05, ***p* < 0.01 vs. the control.

**
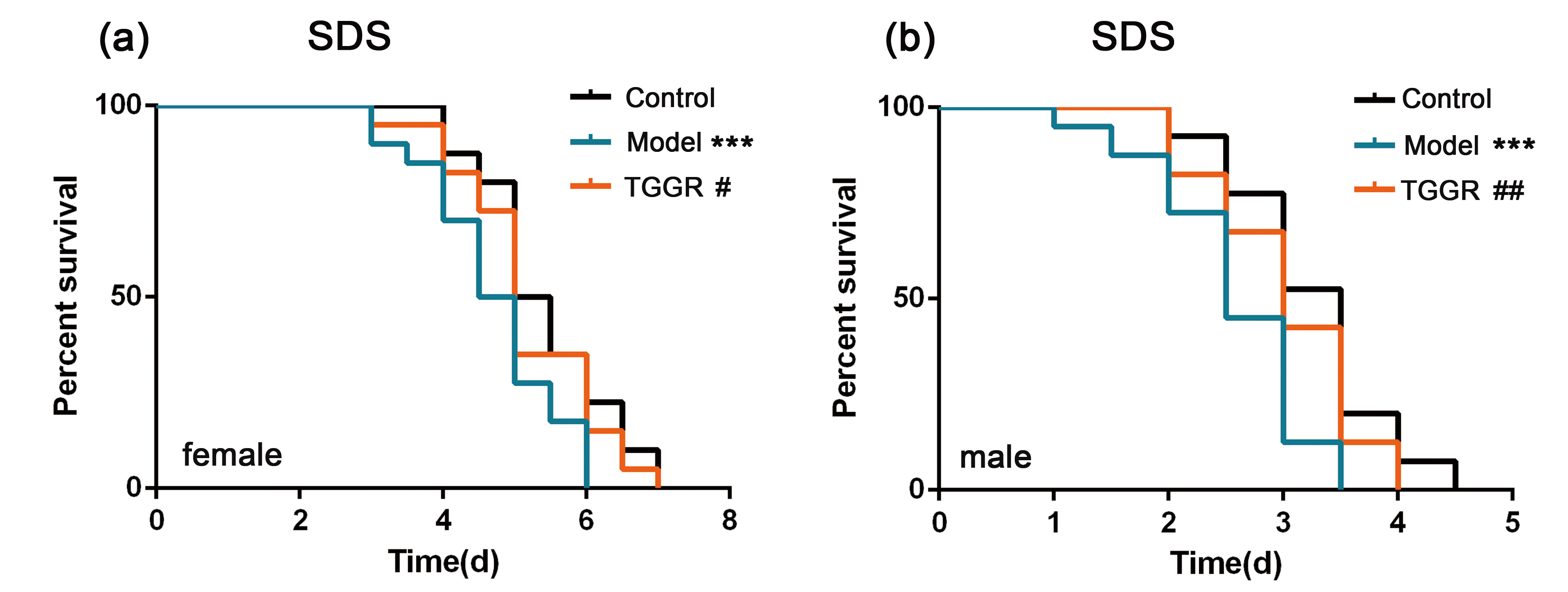
**

**Figure S2.** TGGR can recover SDS damage. Effect of starvation SDS damage on survival time in female (**a**) and male (**b**) with or without TGGR treatment. N = 30 flies in per condition, Log-rank (Mantel-Cox) test. ‘Control’ was 7-day-old flies, ‘Model’ was 40-day-old flies, and ‘TGGR’ was 40-day-old flies with TGGR (5 mg/mL) treatment. ****p*< 0.001 vs. the control; #*p* < 0.05, ##*p* < 0.01 vs. the model; two-tailed unpaired t-test.


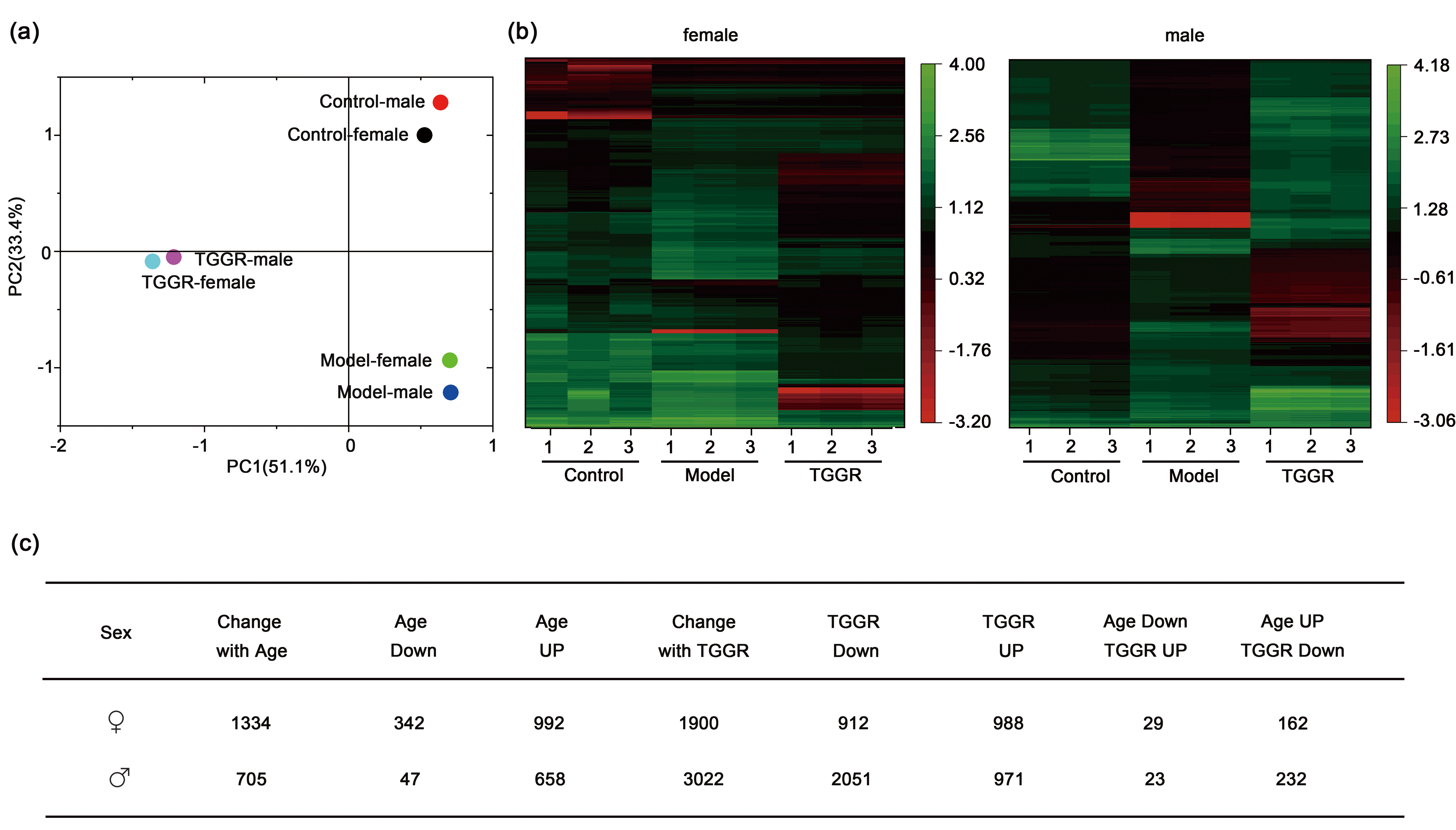


**Figure S3.** Principal component analysis (PCA) and expression clustering profiles of *Drosophila* transcriptomes. (**a**) The Alt-Analyze software was used to compare PCA values for individual RNA-seq transcriptomes isolated from 7 day (control), 40 day (model) to TGGR treatment flies. (**b**) Alt-Analyzer was also used to establish the individual expression clustering profiles for *Drosophila* transcriptome at different ages and treatment conditions. (**c**) Genes with fold changes due to aging and TGGR.


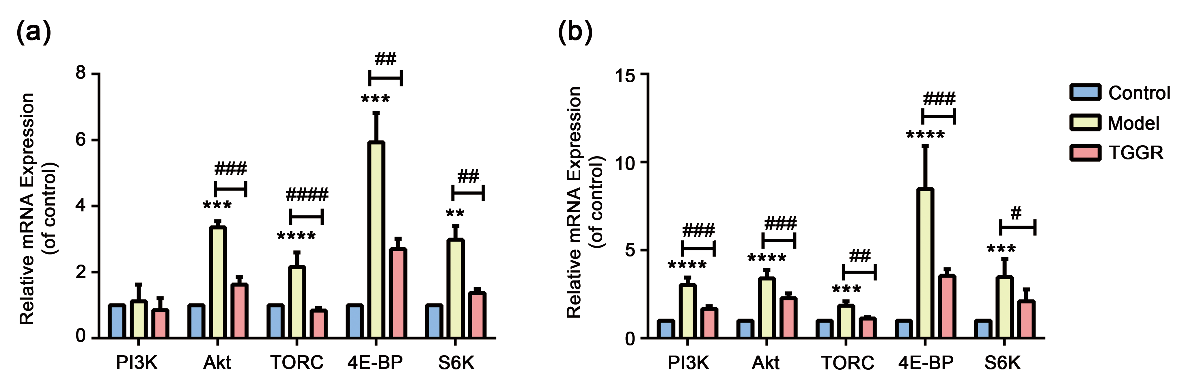


**Figure S4.** Use qRT-PCR to verify gene mRNA levels on the enrichment pathway (**a**: female, **b**: male). (N = 90 flies per condition, two-tailed unpaired t-test). ‘Control’ was 7-day-old flies, ‘Model’ was 40-day-old flies, and ‘TGGR’ was 40-day-old flies with TGGR (5 mg/mL) treatment. ***p* < 0.01, ****p* < 0.001, *****p* < 0.0001 vs. the control; #*p* < 0.05, ##*p* < 0.01, ###*p* < 0.001, ####*p* < 0.0001 vs. the model).


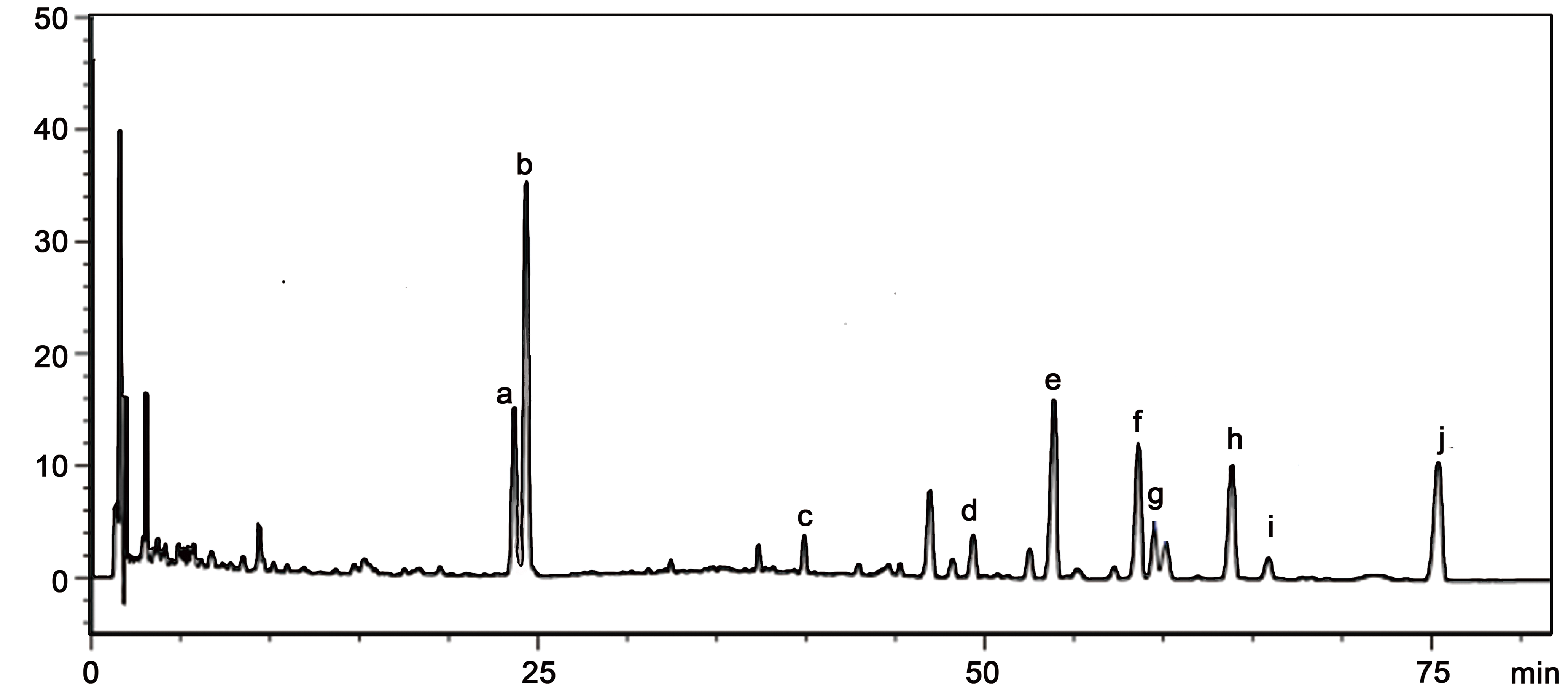


**Figure S5.** The High performance liquid chromatography (HPLC) chromatogram of TGGR. (a: Rg1, b: Re, c: Rf, d: Rg2, e: Rb1, f: Ro, g: Rc, h: Rb2, i: Rb3, j: Rd).
